# BCL::Conf: small molecule conformational sampling using a knowledge based rotamer library

**DOI:** 10.1186/s13321-015-0095-1

**Published:** 2015-09-30

**Authors:** Sandeepkumar Kothiwale, Jeffrey L. Mendenhall, Jens Meiler

**Affiliations:** Department of Chemistry, Center for Structural Biology, Vanderbilt University, Nashville, TN 37232 USA; Department of Pharmacology and Biomedical Informatics, Vanderbilt University, Nashville, TN 37212 USA

**Keywords:** Conformation sampling, Knowledge-based, Fragment-based, Rotamer-library

## Abstract

The interaction of a small molecule with a protein target depends on its ability to adopt a three-dimensional structure that is complementary. Therefore, complete and rapid prediction of the conformational space a small molecule can sample is critical for both structure- and ligand-based drug discovery algorithms such as small molecule docking or three-dimensional quantitative structure–activity relationships. Here we have derived a database of small molecule fragments frequently sampled in experimental structures within the Cambridge Structure Database and the Protein Data Bank. Likely conformations of these fragments are stored as ‘rotamers’ in analogy to amino acid side chain rotamer libraries used for rapid sampling of protein conformational space. Explicit fragments take into account correlations between multiple torsion bonds and effect of substituents on torsional profiles. A conformational ensemble for small molecules can then be generated by recombining fragment rotamers with a Monte Carlo search strategy. BCL::Conf was benchmarked against other conformer generator methods including Confgen, Moe, Omega and RDKit in its ability to recover experimentally determined protein bound conformations of small molecules, diversity of conformational ensembles, and sampling rate. BCL::Conf recovers at least one conformation with a root mean square deviation of 2 Å or better to the experimental structure for 99 % of the small molecules in the Vernalis benchmark dataset. The ‘rotamer’ approach will allow integration of BCL::Conf into respective computational biology programs such as Rosetta.

**Electronic supplementary material:**

The online version of this article (doi:10.1186/s13321-015-0095-1) contains supplementary material, which is available to authorized users.

**Graphical abstract:**
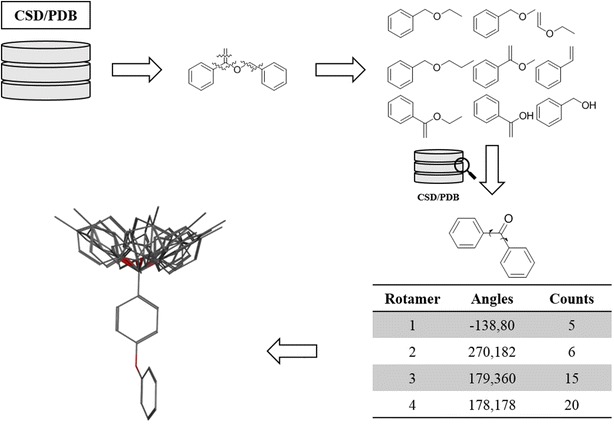
Conformation sampling is carried out using explicit fragment conformations derived from crystallographic structure databases. Molecules from the database are decomposed into fragments and most likely conformations/rotamers are used to sample correspondng sub-structure of a molecule of interest.

## Background

The interactions between small molecules and proteins are important for receptors, transporters, or enzymes to recognize their substrates as well as for small molecule therapeutics to bind to their target protein. The molecular interaction and, hence, the biological function of a small molecule is related to its three-dimensional structure when interacting with the protein. In solution, small molecules are often flexible and exist as an ensemble of conformations in equilibrium with one another. The biologically active conformation may be a single conformation or a small subset from the conformations sampled in solution or a new conformation, induced by protein binding. A uniform sampling of all energetically accessible small molecule conformations is essential for the success of protein small molecule docking simulations [1] for example in structure-based computer-aided drug discovery/design (CADD) [1–3]. However, also ligand-based CADD applications such as three-dimensional quantitative structure activity relationships (3D-QSAR) predictions [4] or pharmacophore modeling [5] rely on the use of conformational ensembles of molecules that capture the bioactive conformation as one of a diverse set of energetically accessible conformations [6, 7].

### Conformational sampling methods

Table 1 summarizes some of the existing conformational sampling methods. Conformation sampling methods can be characterized in several ways. First, the allowed search space can be analyzed: some methods search the entire conformational space, i.e. bond length, angles and torsions can be altered—for example a molecular dynamics simulation in Cartesian space. Other methods restrict the search space to torsion angles only holding bond length and angles fixed. Another approach involves using pre-existing knowledge of small-molecule conformations to restrict the conformational search space even further to likely torsion angles or combinations thereof. Such knowledge-based methods derive torsion angle preferences from molecular mechanics or quantum chemical simulations of small molecules or structural databases like Cambridge Structure Database [8] (CSD) or Protein Data Bank [9] (PDB).

**Table 1:** Conformation sampling methods

Method	Search space	Search strategy	Search method	Scoring function
Caesar [ 39]	Incremental search of torsion angles combined with distance geometry for ring systems	Fragment based	Systematic	CHARMm force field
Catalyst [ 40]	Incremental search of torsion angles with subsequent energy minimization	Non-fragment based	Simulation (MD)	CHARMm force field
Conan [ 41]	Incremental search of torsion angles	Fragment based	Systematic	–
Confab [42]	Incremental search of torsion angles	Non-fragment based	Systematic	MMFF94
Confgen [ 31]	Random walk on energy surface calculated using a truncated version of OPLS_2001	Non-fragment based	Simulation (MC)	MMFFs/OPLs_2001
Corina [ 43]	Knowledge based rules derived from CSD	Non-fragment based	Systematic	Reduced force field for optimizing only ring systems
Enumerated Torsions (*et*) [18]	Incremental search of rule-based torsion angles	Non-fragment based	Systematic	–
Mimumba [ 15]	Incremental search of knowledge-based torsion angles from CSD	Non-fragment based	Systematic	Relative frequency of experimentally observed conformations
Moe (low mode MD) [44]	Constant temperature MD	Non-fragment based	Simulation (MD)	MMFF94
Moe (stochastic search) [22]	Random perturbations of rotatable bonds in increments biased around 30°	Non-fragment based	Simulation (MC)	MMFF94
Moe (Confimport) [22]	Pregenerated fragment conformations obtained from stochastic-search	Fragment-based	Simulation	MMFF94
Moe (systematic) [32]	Incremental search of torsion angles	Non-fragment based	Systematic	MMFF94
Omega [ 33]	Knowledge based torsions from analysis of molecules in PDB and conformations generated by MMFF94	Fragment based	Systematic	MMFF94
RDKit [ 34]	Distance geometry	Non-fragment based	Simulation (distance geometry)	UFF

In addition, it is helpful to single out fragment-based approaches: This search strategy splits a molecule of interest and samples conformations of smaller fragments independently. Candidate conformations of the entire molecule are computed by re-combining constituent fragment conformations. In fragment-based methods, fragments are reused during conformer generation which improves the time-efficiency of sampling. On the other hand, these methods operate on the assumption that all low energy conformations can be created by combinations of low-energy fragments—an assumption that is not always fulfilled.

An alternative classification approach focuses on whether the search space is sampled systematically in its entirety or a search algorithm follows a trajectory that seeks to restrict the search space to low energy conformations. If the conformational space is sufficiently small, systematic approaches can create all possible conformations iteratively and keep all low-energy conformations. An advantage is complete sampling of the entire search space, one disadvantage is slowness. Trajectory-based methods use random or directed perturbations to alter a starting conformation and the resulting conformation is evaluated energetically. In a feed-back loop this energy and possibly derived forces determine the trajectory of the simulation. Molecular dynamics [10, 11], distance geometry [12], genetic algorithms [5], and Monte Carlo [11] (MC) are commonly used simulation methods for the conformational sampling of small molecules.

#### Scoring functions

Most methods score conformations using some form of molecular mechanics energy function. Force field based energy calculations use most frequently the Merck molecular force field (MMFF) [13] or the Chemistry at Harvard Molecular Mechanics (CHARMm) force field [14]. Some methods modify the default versions of these force fields by modifying individual scoring terms or using only a subset of the scoring terms. One alternative approach, as used in Mimumba [15], to scoring small molecule conformations can be derived from knowledge-based scoring functions used in protein structure prediction that analyze the frequency of geometric features observed in structural databases such as the PDB or CSD.

### Knowledge based conformation sampling

Conformations of small molecules can be restricted in terms of commonly seen conformations of constituent fragments in structure databases like CSD. Brameld et al. [16] have shown that conformations of fragments sampled in the CSD are an accurate representation of conformational space seen in drug-like molecules in complex with protein as observed in the PDB. Fragments occur in these structure databases in different chemical environments, leading to them being observed in different conformations. The central hypothesis of this study is that while not all small molecules have been crystallized in all possible conformations, the conformational space accessible to sufficiently small fragments is adequately sampled.

Existing methods like Confect [17] derive torsion profiles for different dihedral bond types from structure databases. Confect treats dihedral bonds as uncorrelated and does not take into account substituent effects. A rule-based proprietary method, developed by Merck research laboratories for internal use, known as *et* for enumerated torsions uses correlated torsion angles to some extent for conformational sampling [18]. The method overlaps multiple fragments containing topologically adjacent rotatable bonds to extend these fragments until they span the entire small molecule. In *et* a proprietary ‘atom typer’ is used to express molecular fragments as unambiguous patterns [19]. The pattern along with associated data for observed torsion angles and frequency constitutes a rule. As of 2001, authors reported that 797 rules had been derived over a period of several years. However these patterns consider only the four atoms involved in a dihedral bond and do not take into account effect of substituents on torsional profile of bonds.

The algorithm BCL::Conf described in the present study goes beyond previous work by using torsional profile of multiple consecutive dihedral bonds and capturing effect of substituents on their torsion profiles. All fragment conformations sampled frequently in the CSD and PDB are considered a knowledge-based ‘rule’ independent of size or number of rotatable bonds. This fragment conformation approach allows BCL::Conf to capture correlations in torsion states for multiple consecutive dihedral bonds in contrast to other methods that treat likely torsion angle states for consecutive bonds in an uncorrelated way. Conformations observed frequently for one fragment are assumed to represent a local energy minimum and are collected in a database. The use of conformations of fragments has also the advantage that these fragment conformations already reside in locally optimal geometries so that only non-local interactions, i.e. clashes, need to be evaluated when fragments are recombined. Lastly, as explicit fragments are used effects of substituents on torsional profiles of rotatable bonds are taken into account. Brameld et al. have shown the effect of substitution on the torsion distribution of common acyclic organic fragments [16].

We expect that the algorithm is therefore particularly tailored for ‘drug-like’ small molecules which are overrepresented in the CSD and PDB databases. BCL::Conf mimics the ‘rotamer’ libraries created to capture amino acid side chain conformations seen in protein structures within the PDB [20] which, ultimately, will ease its integration with protein modeling packages such as Rosetta [21]. BCL::Conf scoring includes a clash score that avoids atom overlap as well as a knowledge-based scoring function that scores conformations based on probabilities of fragment conformations that it contains.

To benchmark BCL::Conf we use a curated dataset containing drug-like ligands found in complex with proteins in the PDB. The “Vernalis generic compound set” [22] has been used in several studies to evaluate the performance of conformational sampling methods enabling a direct comparison of BCL::Conf to other methods [23, 24]. The benchmark study tests for recovery of protein-bound conformation of the ligand and also the ability of BCL::Conf to produce a diverse set of conformations. To remove any bias during benchmarking, the ligands found in the Vernalis dataset were removed from the PDB ligand library. Additionally, ligands were removed from the PDB ligand library if bound to proteins or homologues of proteins present in the Vernalis dataset.

## Implementation

**Fig. 1:**
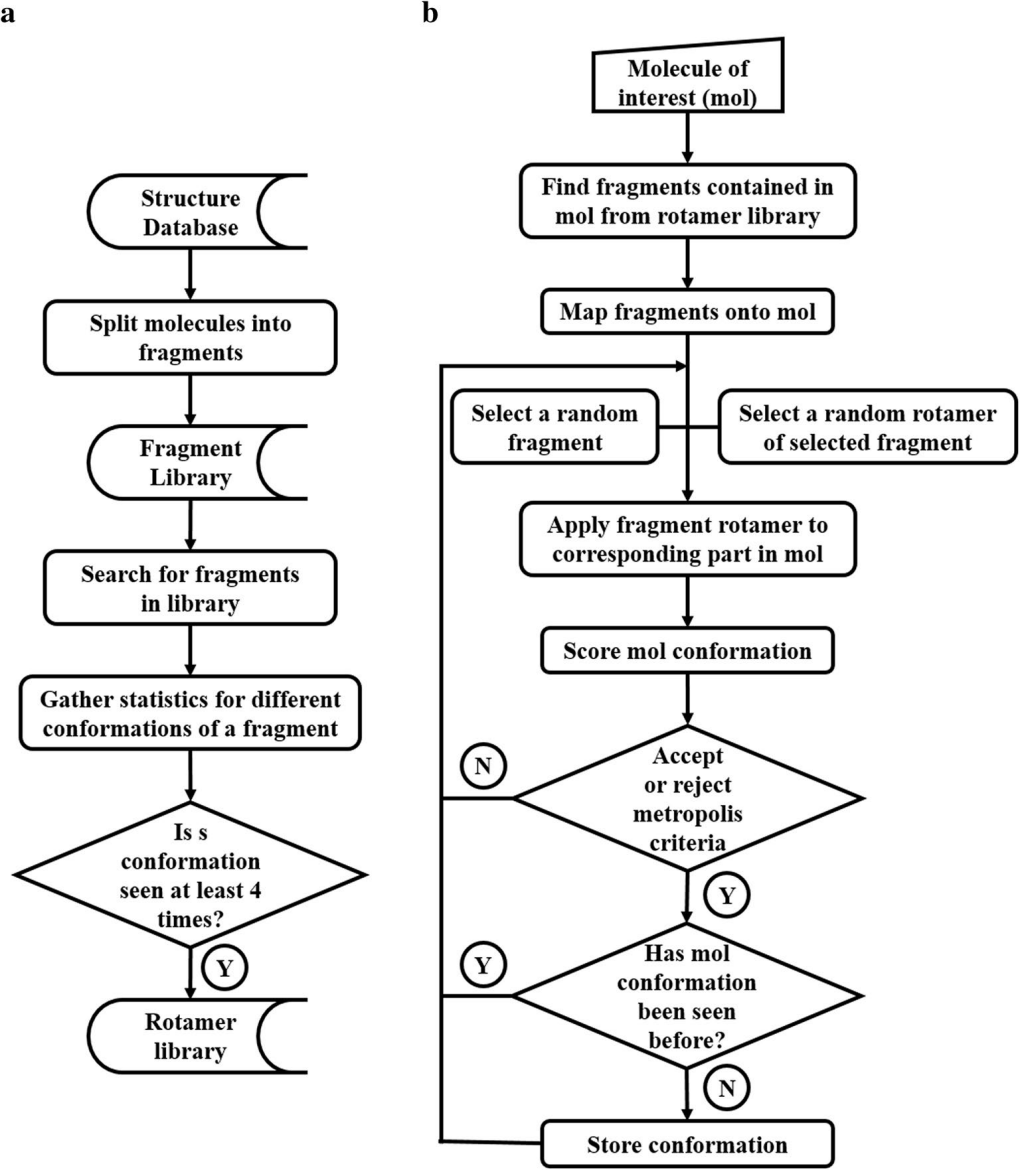
General scheme for BCL::Conf conformation generator. **a** Scheme for generating the rotamer library. **b** Flowchart depicting conformation sampling process. See text for a detailed description

BCL::Conf uses fragments generated from decomposing molecules found in CSD and PDB. For this purpose, non-ring bonds of each molecule are broken iteratively to generate all possible fragments. In a second step all occurrences of one fragment within the structure databases are collected and clustered according to discrete dihedral angle bins. A conformer is then defined as a unique conformation represented as a set of integer numbers, one for each dihedral bond, identifying the bin. This procedure is similar to the definition of ‘rotamers’ that are used to set likely amino acid side chain conformations [20]. A conformer needs to be seen at least four times in the database to be considered a likely conformation of a fragment. It is then added to the rotamer library for sampling. The flowchart for algorithm implemented in BCL::Conf is shown in Fig. 1.

### Fragment library

Small organic molecules from the CSD and PDB were used for generating fragments. The PDB ligands were obtained from the refined dataset in the PDBbind database [25–27]. We removed any molecules for which BCL could not assign correct atom types, molecules with missing 3D coordinates and bad geometries in terms of unrealistic bond-lengths or bond-angles and non-planar aromatic rings or sp^2^–sp^2^ bonds. This resulted in a database containing 113,339 unique molecules. Molecules were broken iteratively at non-ring bonds which generated 56,818,272 unique fragments.

### Rotamer library

**Fig. 2:**
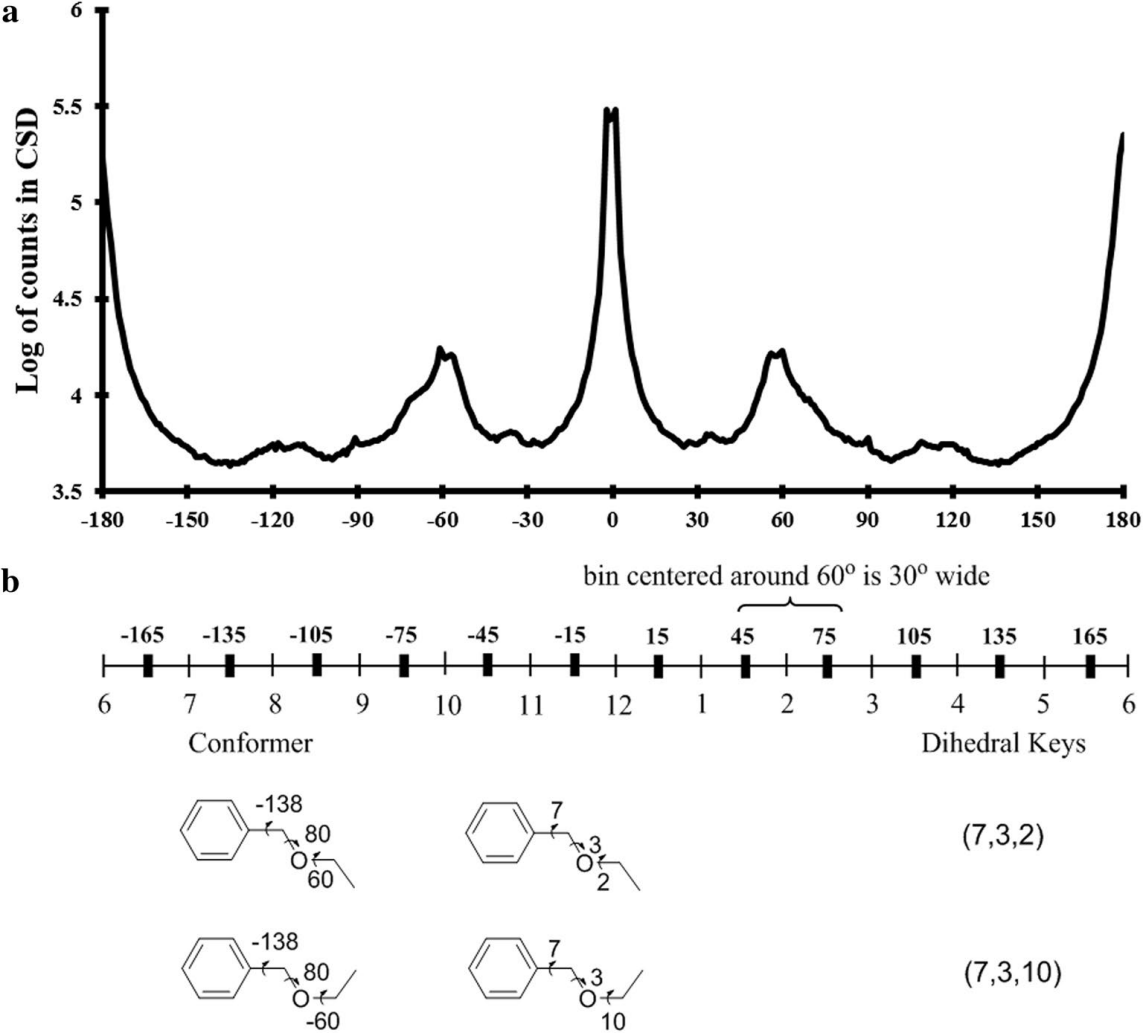
Scheme for torsion angle binning. **a** The *line graph* shows the distribution of dihedral angle measurements of all dihedral bonds over all the molecules in the CSD. **b** Torsional angles are binned into 12 uniform parts with each bin represented as an integer. For example −135 to −165 belongs to bin number 7. Rotamers can thus be represented by a unique key of integers representing each dihedral angle bin

The rotamer library was generated for fragments that are seen frequently in same conformations. A unique fragment rotamer/conformation is identified by a set of integers, one for each dihedral bond. The dihedral bonds of a rotamer are represented as a set of integers depending on the angle measure as explained in Fig. 2. The frequency distribution of dihedral angle measures seen in CSD, shown in Fig. 2, suggests that local minima for dihedral angles occur at canonical values of 0°, 60°, 120°, and 180° and so on. In addition, for certain bond types such as *aromatic*-*chain*-*aromatic* or *aromatic*-*chain*-*any* angles of 90° and 270° are likely (Additional file 1: Figure S1A). Hence, while torsion angles of 90° and 270° are not local maxima when summing over all torsions, they are likely conformations for certain types of torsion angles. Therefore, in order to assign as many likely torsion angles as possible unambiguously and close to a bin center, 12 bins each of which is 30° wide are created centered at 0°, 30°, 60°, 90° and so on. Binning strategies using 30° produces closer to native conformations when 60° binning is used (see “Results and discussion”). All the bonds including the ones that are inside ring systems are described by an integer so that a rotamer can be described as a string of integers. This string is called the bin-signature of a rotamer.

#### Determining dihedral angles

**Fig. 3:**
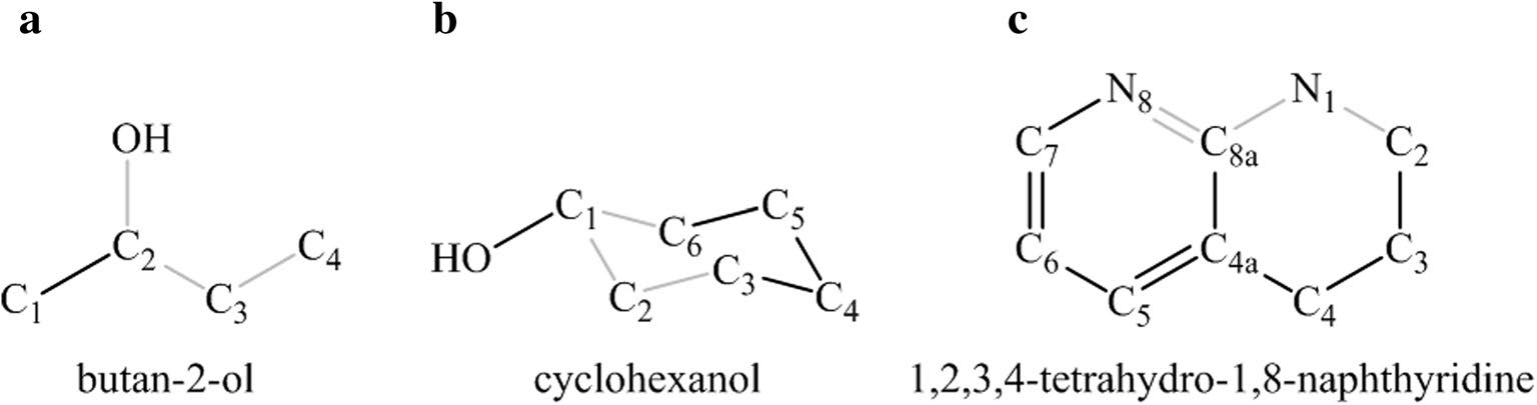
Determination of priority dihedral bonds in molecules. Bond priorities are determined using rules analogous to Cahn–Ingold–Prelog (CIP) rules. In the figure priority dihedral bonds are colored in *grey*. **a** The priority dihedral angle of 2-butanol is c4–c3–c2–O. **b** Priority dihedral bonds in cyclohexanol are defined such that all atoms that define priority dihedral angles are in the ring. Thus for bond C_1_–C_2_, C_3_–C_2_–C_1_–C_6_ is the priority dihedral angle instead of C_3_–C_2_–C_1_–O. **c** For multiple ring systems like 1,2,3,4-tetrahydro-1,8-naphthyridine, priority angles are determined by atom priority using the assumption that all atoms in the multiple ring system are part of one ring. Thus C_2_–N_1_–C_8a_–N_8_ is the priority dihedral angle instead of C_2_–N_1_–C_8a_–C_4a_ as N_8_ is counted to be in the same ring system as the N_1_–C_2_ bond of interest

Since multiple dihedral angles can be measured at each torsion bond, a scheme is required to prioritize which dihedral angle to use and arrive at unambiguous bin-signatures. Therefore a priority dihedral angle is defined. This is accomplished using rules analogous to the Cahn–Ingold–Prelog (CIP) system [28]. For example, as shown in Fig. 3a, 2-butanol has one torsion bond but two dihedral bonds about the single rotatable bond. According to CIP rules, the O–C–C–C dihedral angle will have a higher priority over the C–C–C–C dihedral angle. If out of three possible dihedral angles, two dihedral angles of equally high priority exist, then the third dihedral angle with lowest priority is used. If ambiguity still exists in assigning unique dihedral bonds, for example in the case where all dihedral angles have the same priority, the one with the smallest angle measure is chosen. Priority dihedral bonds in rings are defined in a special way in that all atoms constituting a priority bond are contained in the ring, as shown in Fig. 3b for cyclohexanol. This ensures that for the same ring conformation, a substituted ring system has the same dihedral-signature as an un-substituted ring system. If a fused ring system is present, then priority dihedrals are determined using atom priorities and the assumption that all atoms of the ring system are part of one ring (Fig. 3c). BCL::Conf can identify different ring conformations and use these in conformational sampling. Since dihedral angles are assigned in a unique way for a molecule of interest, a unique rotamer of the molecule has a unique dihedral bin signature. Table 2 shows different rotamers for a fragment from the rotamer library and their bin signatures.

**Table 2:** The rotamers of a fragment from the rotamer library


Rotamer #	Bond1	Bond2	Bond3	Bond3	Bond4	Bond5
1	6	5	6	12	2	6
2	6	3	6	12	5	6
3	6	1	6	12	2	6
4	6	5	6	12	4	6
5	6	1	6	12	5	6
6	6	5	6	12	5	6
7	6	4	6	12	2	6
8	6	1	6	12	1	6
9	6	5	6	12	1	6

Five rotatable dihedral bonds are labeled in the figure and for each rotamer, the dihedral bins are shown

#### Searching rotamers

In building the rotamer library, all instances of every fragment are collected in the molecular database using a graph isomorphism search [29]. For each fragment, all unique rotamers are identified using dihedral bin signatures. Then statistics is gathered for each rotamer including rotamer counts, i.e. the number of times a rotamer is seen in the database, and dihedral angle statistics, i.e. the average angle measure and standard deviation of dihedral bonds within each bin. A representative structure for a fragment is obtained by clustering all instances of the most frequently observed rotamer in the structure database on the basis of root mean square deviation (RMSD) after superposition. In addition, if a fragment contains a ring in different conformations, explicit coordinates are stored for each rotamer. A conformer is added to the rotamer library of a fragment if it is seen at least four times in the structure databases (combined CSD and PDB), i.e. it can be considered a likely conformation for that fragment. A total of 231,049 fragments are observed that have at least one conformer which is seen at least four times in the molecular database and hence these fragments are retained in the rotamer library. Table 3 shows the rotatable bond distribution and rotamer distribution of fragments in the rotamer library.

**Table 3:** (a) Rotatable bond distribution in the rotamer library, (b) conformation statistics in the rotamer library

Number of rotatable bonds	Number of fragments
0	47,205
1	38,616
2	31,225
3	20,500
4	15,221
5	13,665
6	14,014
7	14,693
8	14,435
9	13,492
≥10	10,064

Number of rotamers	Number of fragments
1–5	219,684
6–10	10,840
11–15	1768
16–20	488
21–25	209
26–30	82
31–35	47
36–40	18
41–45	8
46–50	1
>50	3

### Search fragments from the rotamer library that are contained in the molecule of interest

**Fig. 4:**
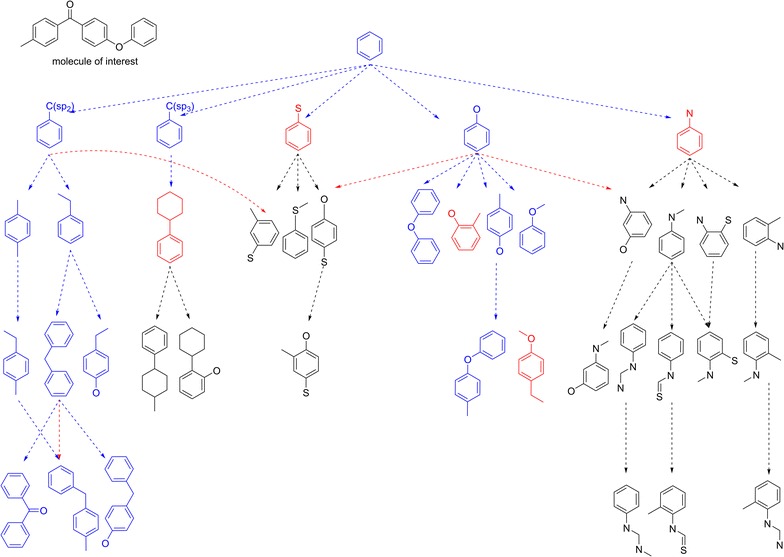
Graph database for storing rotamer library for fast searching. The figure illustrates a rooted graph layout of fragments where each node is a unique constitution. The child nodes originating from the root are such that the root (in this case, benzene fragment) is their immediate substructure among all the fragments shown in the graph. Fragments contained in the molecule of interest are colored in *blue* while those that are not are in *red* or *black*. For fragments in *black* no substructure search is performed because their parent fragments were not found in the molecule of interest. The edges represent all possible search paths for finding fragments contained in the molecule of interest. Paths in *blue* are the actual searches that were performed for finding fragments for the query molecule. Paths in *red* and *black* are never taken during the search. *Red* colored paths are redundant search paths that have already been covered in a previous search

Conformational sampling begins with searching fragments contained in a molecule of interest. This involves substructure searches to identify all suitable fragments in the rotamer library. A hierarchical search has been implemented to minimize the number of substructure searches. The rotamer library is represented as multiple rooted graphs where each node is a unique constitution. The root nodes are not contained in any other fragments. Child nodes are such that the parent node is an immediate substructure. Figure 4 illustrates a rooted graph with benzene as root. Benzene is an immediate substructure of its child nodes i.e. toluene-like fragment which is an immediate substructure of cyclohexylbenzene-like fragment.

The fragment searching begins at the root node of graphs. If the root node is contained in the query molecule, all its immediate child nodes will be searched to determine if they are contained within the molecule of interest. For all child nodes contained, their immediate child notes are considered and so on. In Fig. 4 fragments that are part of molecule are colored in blue—i.e. a successful substructure search. Fragments colored red indicate that a substructure search was performed but unsuccessful. This terminates further searches in this branch of the tree. Fragments colored in black are not considered for a substructure search, because their parent fragments were not contained within the molecule of interest (colored red). The edges in the graph are directed from parent to child nodes and represent search paths that can be taken to find all constituent fragments in a query molecule. Paths in blue color are actual paths that are taken to identify all the fragments contained in the molecule interest while the paths in red or black are never explored. Search paths in black originate from fragments that are not contained with the molecule. Red paths represent redundant searches in the tree. This hierarchical tree structure of the data enables fast and efficient searching of all the fragments contained within a molecule of interest.

### Generation of initial 3D structure from minimum set of fragments with most likely conformation

An initial 3D conformation is necessary for using the conformer sampler implemented in BCL::Conf. The BCL software suite accepts molecules in the MDL [30] format. A 3D structure generator has been implemented to generate an initial 3D structure if coordinates are not provided. BCL::Conf can generate starting coordinates from connectivity information provided in the MDL format. When coordinates or 3D structure is not available, BCL::Conf first searches for all fragments from the rotamer library that are contained in a molecule of interest. The algorithm identifies the minimum number of fragments that can be connected to generate molecule of interest. The most likely conformers of fragments are then connected to assemble the molecules of interest and generate an initial 3D structure which may or may not have clashes between atoms. As this conformation only serves as a starting point with the objective to place all torsion angles into a locally reasonable conformation and is not necessarily part of the output ensemble of conformations, atom clashes are not a problem.

### Monte-Carlo Metropolis sampling for efficient search of conformational space for likely non-clashing conformations

Conformational sampling begins by identifying fragments from the rotamer library that are contained in the molecule of interest whose conformations need to be sampled. From the fragments contained in the molecule of interest, a random one is selected and one of its rotamers is applied to change the conformation of the molecule. The rotamer is selected based on probability of its occurence in the structure database (Fig. 1b). If the chosen fragment rotamer contains a different ring conformation, then the whole molecule is reassembled by using the chosen conformer as the starting fragment. By default only a subset of rotamers that are observed most frequently are used in sampling. The cutoff value is specified at half of the probability of the most likely rotamer. If more sampling is desired, an option to use the full rotamer set can be specified at the command line.

Starting with the input structure of the molecule of interest, new conformations are created in a continuous MC trajectory. A MC step is accepted or rejected based on the Metropolis criterion. The energy or score used is a combination of atom clashes and propensity of observing constituent fragment rotamers in structure database. The atom clash score is calculated by evaluating non-bonded atom pairs for clashes using Eq. 1.

1 $$Atom\,\,Clash\,\,Score = \frac{{\mathop \sum \nolimits_{i > j} 2*score_{{atom_{j } }} \left\{ {\begin{array}{*{20}l} {0, \,\;dist \ge cov} \\ {1, \;dist \le cov} \\ \end{array} } \right.}}{Number\,\,of\,\,atoms\,\,in\,\,the\,\,molecule}$$

where $dist\mathop{=} \limits_{}^{\text{def}}$ distance between non-bonded atoms *i* and *j*, $cov\mathop {=} \limits_{}^{\text{def}}$ sum of covalent radii of atoms *i* and *j.*

Rotamer propensity score (Eq. 2) leverages the statistics on the rotamer of a particular fragment to estimate the likelihood of a particular conformation. The hypothesis is that there is a correlation between frequency of occurrence and free energy of a fragment conformation. For a given molecular conformation, the observed rotamer of each of the constituent fragments is determined. The observed rotamer propensity for a fragment is calculated by dividing observed rotamer count by average rotamer counts. The overall conformation score is obtained by summing up observed rotamer propensities of all the constituent fragments. If, for a fragment none of the rotamers are seen in a given conformation, then a pseudo rotamer count equal to half of the least common rotamer count is used instead. The propensity score is normalized by dividing it by absolute value of maximum possible propensity score for the molecule of interest.

2 $$Propensity \;Score = {{\mathop \sum \limits_{i = 0}^{N} \left( { - ln\frac{{R_{i} \times F_{i} R_{j} }}{{\mathop \sum \nolimits_{j} F_{i} R_{j} }}} \right)} \mathord{\left/ {\vphantom {{\mathop \sum \limits_{i = 0}^{N} \left( { - ln\frac{{R_{i} \times F_{i} R_{j} }}{{\mathop \sum \nolimits_{j} F_{i} R_{j} }}} \right)} {\mathop \sum \limits_{i = 0}^{N} \left( {ln\frac{{R_{i} \times F_{i} R_{max} }}{{\mathop \sum \nolimits_{j} F_{i} R_{j} }}} \right)}}} \right. \kern-0pt} {\mathop \sum \limits_{i = 0}^{N} \left( {ln\frac{{R_{i} \times F_{i} R_{max} }}{{\mathop \sum \nolimits_{j} F_{i} R_{j} }}} \right)}}$$

where $N\mathop {=} \limits_{}^{\text{def}}$ number of fragments that are part of the molecule of interest, $F_{i} \mathop{=}\limits_{}^{\text{def}}$*i*th fragment of molecule, $R_{i} \mathop{=}\limits_{}^{\text{def}}$ number of rotamers of the *i*th fragment, $R_{max} \mathop{=}\limits_{}^{\text{def}}$ counts of the most common rotamer, $F_{i} R_{j} \mathop{=}\limits_{}^{\text{def}}$ counts of *j*th rotamer of the *i*th fragment.

## Results and discussion

We assess the performance of BCL::Conf (BCL) with curated generic ligand dataset known as the Vernalis dataset [22], in comparison with Confgen [31], Moe (Confimport) [32], Omega [33] and RDKit [24, 34]. The first metric defined as the completeness criteria is the fraction of molecules for which any conformation was generated. The second comparison is the ability of the method to produce ligand conformations within a specified RMSD value to the native conformation of ligands in protein–ligand complexes. This analysis is reported as the percentage of molecules whose conformations are recovered within a given threshold RMSD value. The third criteria for comparison is diversity, that is how similar or different are the generated conformations. Finally a comparison of the methods on computational speed is provided. We also report results for different flavors of BCL that use different schemes for rotamer library generation—(a) using a 60° torsion binning (BCL_60), (b) rotamer library derived from only the CSD (BCL_CSD), (c) rotamer library containing only single dihedral bond torsion profiles (BCL_D).

Conformational sampling with different methods was performed to yield a symmetry corrected RMSD diversity of 0.25 Å—i.e. no two conformations have a RMSD smaller than 0.25 Å—and a maximum of 100 conformers per molecule.

### Ligand dataset

Vernalis dataset is used here to compare BCL::Conf to other existing methods in the field. The Vernalis Dataset (Additional file 2), compound set introduced by Chen and Foloppe [22–24], contains 253 ligands derived from high-resolution protein–ligand complexes found in the PDB and includes the Bostrom [35, 36] ligand set and Perola [37] ligand set. The Vernalis Dataset has been used in previous benchmark studies to compare Moe, Catalyst and Confgen methods for conformation sampling [22–24].

### Conformer generation methods

BCL::Conf (BCL): Conformation sampling was carried out by providing ligands in the MDL format with all atom coordinates set to zero to remove any initial conformation bias. The rotamer library uses the 30° torsion binning scheme to determine dihedral keys. It is derived from the CSD and the refined set of PDBbind database minus the Vernalis dataset ligands to remove any bias. Conformers were generated in 200 iterations of MC fragment sampling at a temperature of 3.0 such that they were at least 0.25 Å away from each other. Table S2 (see Additional file 1, Additional file 3) shows parameter optimization for native conformer recovery in terms of RMSD with different temperature and iteration values. The row shaded in gray corresponds to parameters used for comparing to other methods.

BCL_60: Conformations were sampled using the same settings as described for BCL::Conf except that 60° torsion binning was used instead of 30°. This experiment tests the effect of 60° binning on conformation sampling.

BCL_CSD: Same parameters as used for BCL:Conf with the only difference being that the rotamer library was sourced from only the CSD. This experiment shows the effect of adding PDB fragment conformations.

BCL_D: Conformation sampling was performed by using torsion angle statistics for single dihedral bonds derived from molecules in the CSD and PDBbind databases. Fragments containing only four atoms and a single dihedral bond from the rotamer library were used for this experiment—i.e. the smallest possible fragments. This experiment tests the impact of the addition of larger fragments that sample the correlation between multiple torsion angles. Initial conformation bias in benchmark dataset molecules was removed by perturbing all dihedral angles to random values. The conformers were generated using the same set of parameters as that for BCL.

Confgen: Confgen systematically samples rotatable bonds, ring conformations, nitrogen atom inversions and amide bond conformations. Force field OPLS_2001 is used for calculating potential for rotating about each rotatable bond [31]. In the present study, conformer generation was done starting from SMILES string of ligands in the Vernalis dataset. SMILES string were generated using Maestro from the dataset ligands in MDL format. Confgen has been reported to reproduce 93 % of molecules within 1.5 Å in the comprehensive mode [31]. 250 conformers were generated with Congen in the comprehensive mode by keeping RMSD cutoff at 0.25 Å, energy cutoff at 104.6 kJ/mol (default value). 100 conformations were saved per ligand for comparison.

Moe-conformation_import (Moe): Conformational import is a high-throughput conformer generation method in Molecular Operating Environment (Moe). Molecule of interest is divided into overlapping fragments and these are searched in a pregenerated library of fragment conformations. If a fragment is not found, conformations are generated using a stochastic conformation search algorithm available in Moe. For this study, the Vernalis dataset was provided such that all atom coordinates were set to zero. The default parameters specified with Moe have been determined to perform best in previously reported benchmark studies [22–24]. The MMFF94x force field and Generalized Born solvation model was during ligand conformation generation. Fragment conformation energy cutoff was kept at a default of 4 kcal/mol. The program was constrained to maintain stereochemistry of the input structures but allowed to sample ring conformations. The stochastic search protocol that conformation import uses for creating conformations of fragments missing in database was modified to generate fragment conformers that were 0.25 Å apart in RMSD. Fragment conformations that were within 15 kcal/mol window of the lowest energy conformer were retained for the stochastic search.

Omega: Omega is a systematic knowledge based conformer generator developed by Openeye Scientific Software. It exhaustively enumerates all rotatable torsions using a knowledge-based list of angles which are then sampled by geometric and energy criteria [33]. The torsion library is derived from analysis of a set of experimental crystal structures from the PDB and from energy scans of torsions against MMFF94. Default parameter values were used except RMSD and MaxConfs which was set to 0.25 and 100 respectively to specify custom conformation diversity level and limit the number of output conformations.

RDKit: RDKit uses distance geometry algorithm described by Blaney et al. for sampling ligand conformations [38]. A distance bound matrix is calculated for a molecule of interest based on connection table and a set of rules. The matrix is smoothed using a triangle-bounds smoothing algorithm. Random distance matrices that satisfy the bounds matrix are generated followed by embedding in 3D dimension to generate conformations. In a final step, embedded coordinates are cleaned up using a crude force field and the bound matrix [23]. In this study, ligand conformations generated using RDKit were minimized using the Universal Force Field ‘uff’ as suggested by Ebejer et al. [24]. 100 conformations were generated followed by minimization and pruning to remove conformations that measure less than 0.25 Å away from each other in RMSD.

#### BCL::Conf generates conformations for all drug-like small molecules

While BCL, Confgen, Moe and RDKit are able to generate conformations for all the molecules of the Vernalis dataset, Omega could not for 16 molecules due to missing fragments in its library.

#### Recovery of experimentally observed conformations

**Fig. 5:**
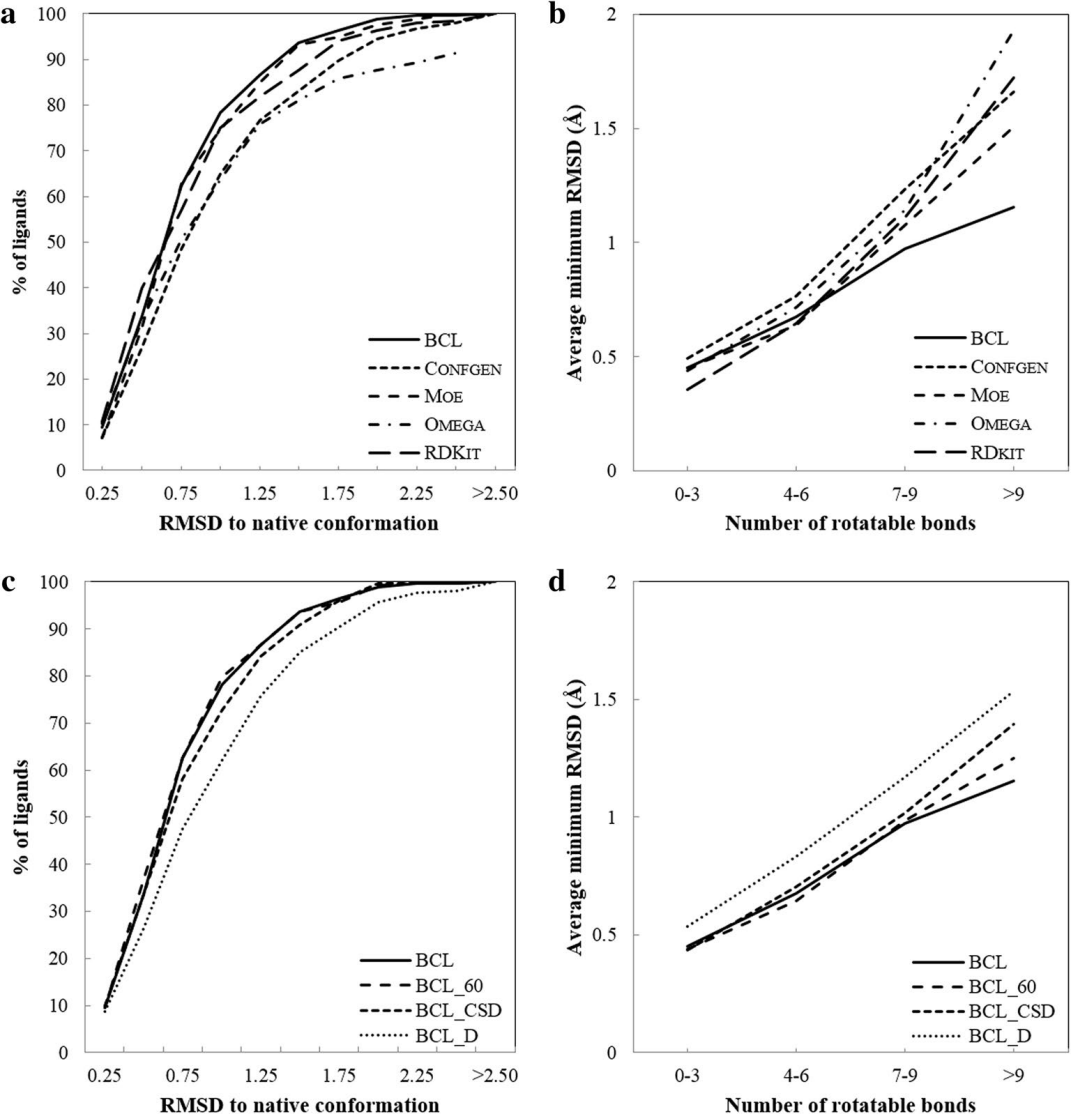
Benchmarking results for the Vernalis dataset. **a** The plot represents percentage of ligands (y-axis) for which different methods produce at least one conformer within an RMSD value less or equal to the RMSD value on the x-axis. **b** Quality of conformations sampled as the number of rotatable bonds increases. The average RMSD of conformers closest to native structure is plotted on the y-axis as the number of rotatable bonds increases (x-axis). **c** Same as **a** for different rotamer libraries used. “BCL” refers to recovery using 30° dihedral bins with rotamers derived from both the CSD and PDB. “BCL_CSD” leverages conformations from the CSD only. “BCL_D” refers to experiments in which instead of fragments containing multiple torsion angles, statistics on single dihedral angles were used for sampling conformations. “BCL_60” refers to a rotamer library that uses 60° dihedral bins are. **d** Quality of conformations sampled as the number of rotatable bonds increases for BCL, BCL_CSD, BCL_D, and BCL_60

The native conformation recovery by BCL, Confgen, Moe, Omega and RDKit is plotted in Fig. 5a. Figure 5a shows the percent recovery of native conformation of ligands at different RMSD cutoff values. BCL recovers native conformer for 11 % of ligands within 0.25 Å, 79 % within 1.0 Å and 99 % within 2.0 Å. Figure 5c shows the effect of rotamer library source (CSD; single dihedral torsion profiles; and CSD + PDB) and binning strategy (30° or 60°) on conformation recovery. Conformation recovery is slightly lower when fragment rotamers observed in only the CSD are used suggesting unique rotamers or significant deviation from canonical values that are observed in ligands bound to proteins. Recovery is not effected significantly when 60° bins are used.

**Fig. 6:**
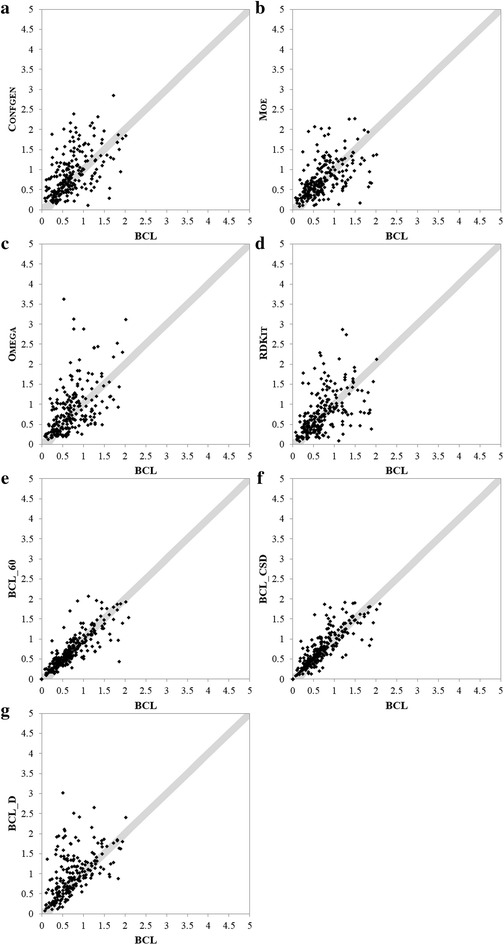
Pair-wise comparison of BCL::Conf to other methods. **a**–**g** Plot the RMSD to native for the BCL on the x-axis, for other methods or flavors of the BCL on the y-axis. BCL::Conf samples closer to native conformations for points that lie above the diagonal. Conformations plotted within the *shaded region* differ by less than 0.25 Å

Figure 6 shows pairwise comparison of Confgen, Moe, Omega, RDKit, BCL_60, BCL_CSD and BCL_D to BCL in generating conformer closest to native. Each point corresponds to a molecule in a test set. The coordinates of a point corresponds to the RMSD of closest to native conformer generated by BCL (x-axis) and the method being compared (y-axis). Molecules for which closest to native conformation generated by the pair of methods is within 0.25 Å RMSD of each other are plotted in shaded gray area. For points above the shaded region, BCL recovers lower RMSD conformer compared to the other method referenced. The molecules for which Omega could not generate conformations are omitted from the graph and statistical analysis when comparing to BCL.

Figures 5 and 6 suggest that BCL is better than other methods and other flavors of BCL being compared. Wilcoxon Matched-Pairs Signed-Ranks statistical test was performed to compare conformations generated by BCL to those produced by other methods for each molecule in the Vernalis dataset. The statistics test was performed using R software package. BCL generated closer to native conformations compared to Confgen, Moe, Omega and BCL_D at p value <0.01 over all the molecules. When compared to BCL_CSD, BCL generates more native like conformations at p value <0.05. Statistically there is no significant difference in native recovery between BCL, BCL_60 and RDKit. However, 30° binning allows recapitulation of frequently observed 90° or 270° rotamers of dihedral bonds containing *aromatic*-*single*-*aromatic* or *aromatic*-*single*-*any* (Additional file 1: Figure S1B).

#### Effect of the number of rotatable bonds on native conformation recovery

Figure 5b, d show the average RMSD of closest to native conformation of molecules plotted against number of rotatable bonds. Figure S2 (Additional file 1) plots the average number of conformations generated by different methods for molecules of different rotatable bonds. BCL is better than other methods at producing closer to native conformers for molecules with greater than six rotatable bonds as suggested by Wilcoxon Paired test at p value <0.05. For molecules containing four to six rotatable bonds, BCL performs better than Confgen and Omega respectively at p value <0.01. There is no significant difference between quality of conformations generated between BCL, Moe and RDKit for molecules with up to six rotatable bonds. For different flavors of BCL, there is no significant difference between BCL and BCL_60 in native conformation recovery based on rotatable bonds. However, statistical analysis clearly shows that using extended fragments improves native conformation recovery compared to using single dihedral bond statistics (BCL_D) for molecules greater than three rotatable bonds at p value <0.01. BCL produces closer to native conformations compared to BCL_CSD for molecules with greater than 10 rotatable bonds.

#### Diversity of conformational space sampled

**Fig. 7:**
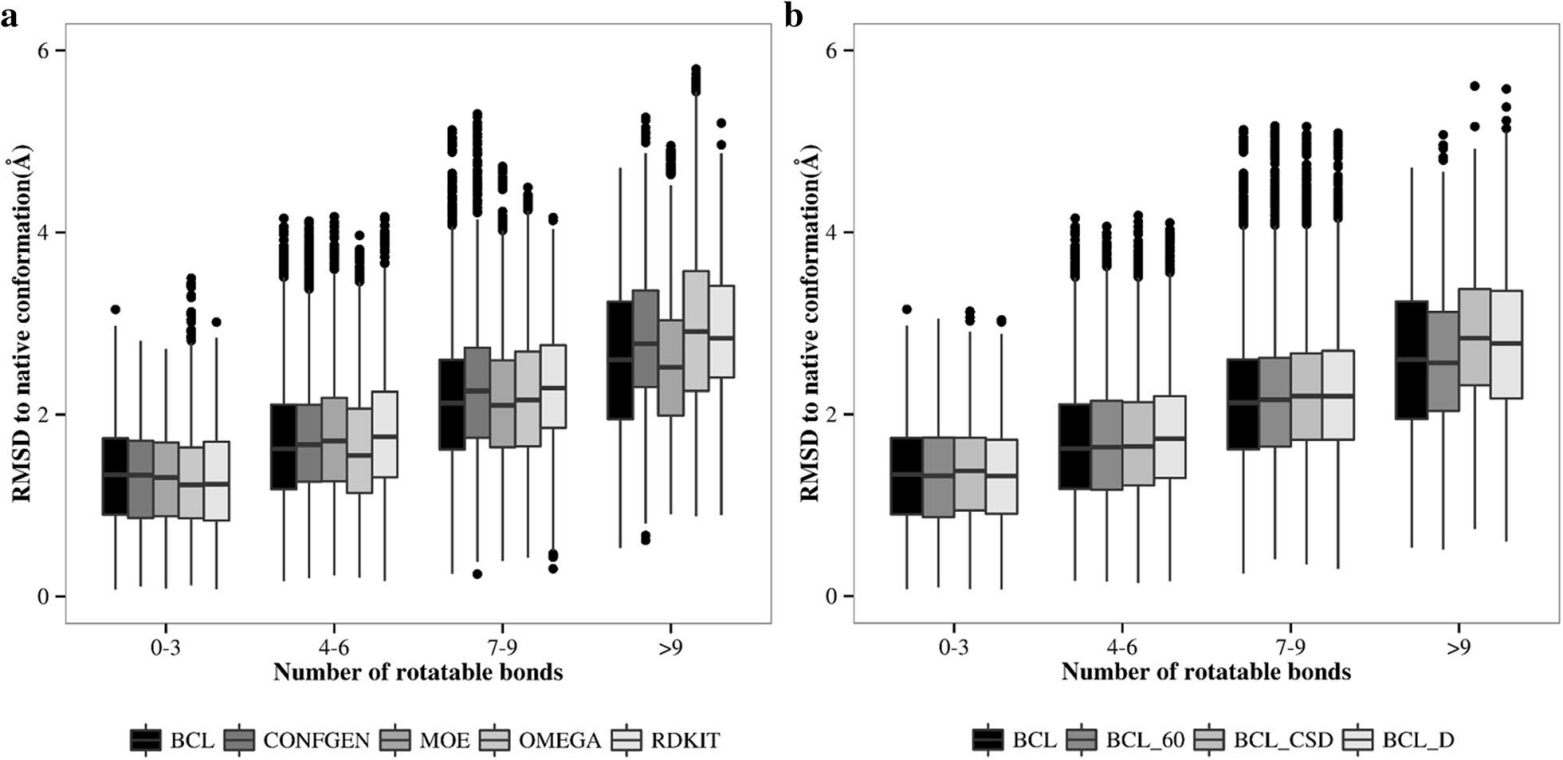
The *box plots* show the diversity of generated molecular conformations depending on the number of rotatable bonds. The *upper* and *lower edges* of *box* correspond to the first and third quartiles. The *horizontal dash* in the *box* represents the median value. The *whiskers* extend from edge to highest/lowest value that is within 1.5 × Inter-Quartile (IQR) of the *box*, where IQR is the distance between the first and third quartile. **a** Conformation diversity produced by different methods, **b** conformation diversity obtained by using different flavors of BCL

Diversity of ligand conformations is an important consideration for ligand docking studies. A representative sample that covers ligand’s sample space is therefore desired. Figure 7a, b show the distribution of RMSDs of conformers against the number of rotatable bonds. Box plots show the distribution of conformer RMSD with respect to native structure. The upper and lower edges of box correspond to the first and third quartiles. The whiskers extend from edge to highest/lowest value that is within 1.5 × Inter-Quartile Range (IQR) of the box, where IQR is the distance between the first and third quartile. The data beyond whiskers are plotted as outliers. The horizontal dash in the box represents the median value. Diversity of conformations generated by all the methods is comparable. Confgen, Moe and RDKit sample conformations more efficiently compared to BCL for molecules with up to three rotatable bonds (see Additional file 1: Figure S2). The reason is that smaller fragments have large number of rotamers with similar energy profiles. Larger fragments on the other hand have fewer local minima allowing sampling of relevant conformations in fewer steps.

#### Comparison of CPU time requirements

The computational run time for the different methods except Omega was compared on Intel Xenon model 26 running at 3.2 GHz with 24 Gb of RAM. All the methods take less than 2 Gb of RAM. BCL generated conformations for a single molecule in 1.6 s compared to 1.9 s taken by Confgen, 5.1 s for Moe, 0.5 s for Omega and 10.2 s for RDKit. Computation time of when using only dihedral torsion profiles i.e. BCL_D is 0.7 s/molecule.

## Conclusions

We have developed a conformational search method called the BCL::Conf and validated it against other methods in the field like Confgen, Moe, Omega and RDKit. The method utilizes the conformational space seen in the structure databases, CSD and PDB, to sample conformations of small-molecules. BCL::Conf is compared to other methods in three measures which are critical in computational drug discovery process: (a) the ability to generate conformation close to experimentally observed structure, (b) diversity of conformations indication coverage of sample space of molecules, (c) performance in terms of speed. The benchmark study was performed using a curated dataset of high resolution X-ray crystal structures from the PDB, Vernalis datasets, containing 253 molecules.

BCL::Conf is capable of reproducing bioactive conformations generating conformers that are structurally close to experimentally determined structures. Analysis of coverage space shows that BCL::Conf generates a diverse set of conformers performing as well as Moe and RDKit, however in much shorter time. BCL:Conf is better and more efficient in sampling molecules with greater than three rotatable bonds as indicated in Fig. 5b and Figure S4 (Additional file 1). Using extended fragments gives BCL:Conf a distinct advantage over other methods in sampling more flexible molecules efficiently. The study shows utility of using explicit fragment conformations to recapitulate protein-bound ligand conformations. A slightly reduced performance is seen when using rotamers derived from only the CSD (Fig. 5c). The somewhat reduced accuracy could result from biases in the fragment sets between CSB and PDB or biases in dihedral angles between ligands bound to proteins and ligands residing in a crystal. Nonetheless results reported in this paper suggest that fragment conformations obtained from the CSD seen in structure databases can be used to adequately model small molecule conformations bound to proteins.

BCL::Conf extends the idea of protein side-chain conformer sampling to fragments of small molecules. The method is novel as it takes into account torsion correlations and substituents effects on fragment torsion profiles. It has been designed and developed to be integrated with RosettaLigand which is part of the macromolecular modeling suite Rosetta.

## Availability and requirements

- Project name: BioChemicalLibrary
- Project home page: http://meilerlab.org/bclcommons
- Operating system(s): Supported on Linux, Apple and Windows
- Programming language: C++
- Other requirements: Access to current CSD license (individual or institutional)
- License: Open source with restrictions, See http://meilerlab.org/bclcommons/license
- Any restrictions to use by non-academics: commercial license needed

## Additional files

**Additional file 1.** Supplementary data and protocol capture describing steps to reproduce data.
**Additional file 2.** The vernalis dataset molecules without 3D coordinates.
**Additional file 3.** protocol capture files mentioned in Supplement.docx.

## Abbreviations

CADD: computer aided drug discovery/design
CHARMm: Chemistry at HARvard molecular mechanics
Moe: molecular operating environment
CSD: Cambridge structure database
MC: Monte Carlo
MMFF: Merck molecular mechanics force field
PDB: protein databank
RMSD: root mean square deviation
3D-QSAR: three-dimensional quantitative structure activity relationships
