# Supplementary material for: BCL::Conf: small molecule conformational sampling using a knowledge based rotamer library
Source: J Cheminform. 2015 Sep 30;7:47. doi: 10.1186/s13321-015-0095-1 (PMC4607025; doi:10.1186/s13321-015-0095-1)
Supplement: Supplementary file 1 — Additional file 1. Supplementary data and protocol capture describing steps to reproduce data. [file 13321_2015_95_MOESM1_ESM.docx]

# Supporting information

Figure S1


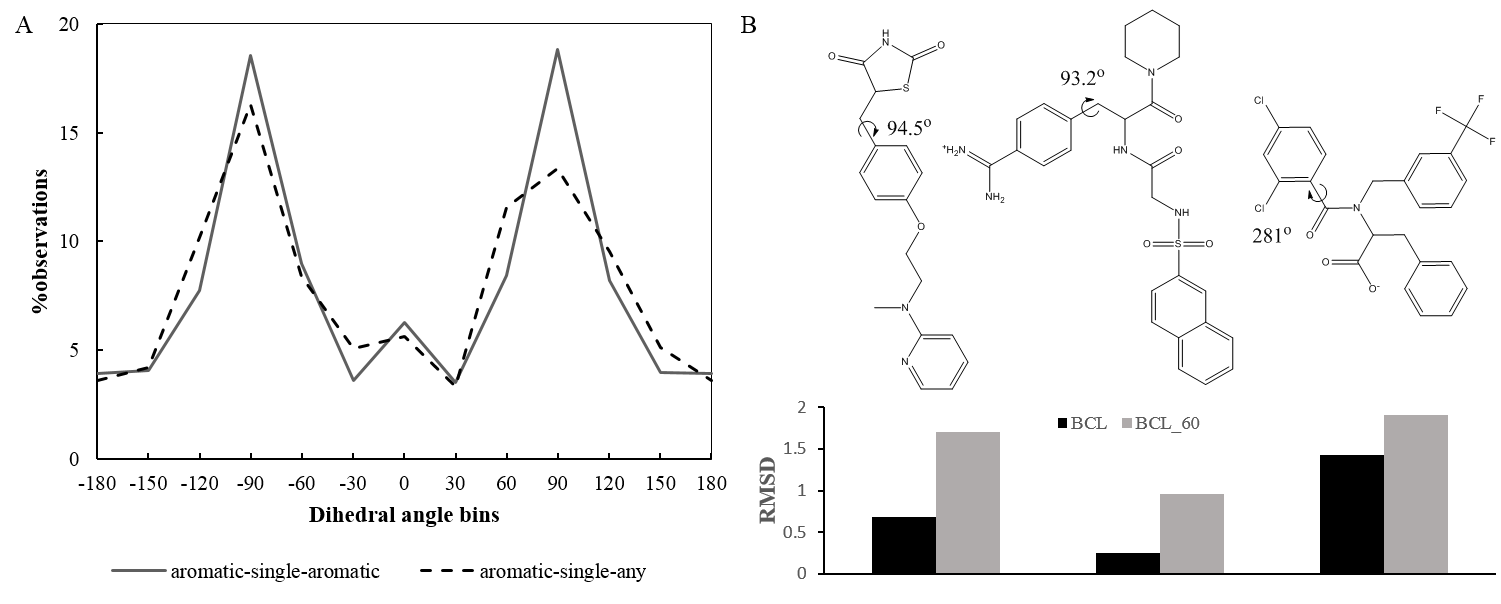


Figure S1. Propensity of certain dihedral bonds containing a sequence of aromatic-single-aromatic and aromatic-single-any bonds to measure 90° or 270°. A) The figure shows distribution for seven aromatic-single-aromatic and 129 aromatic-single-any bonds for which there are at-least four 90° or 270° rotamers and are more abundant compared to 30° or 60° rotamers. Average observation of rotamers is plotted for dihedral bonds is plotted. B) Molecules from the Vernalis dataset containing aromatic-single-single bonds measuring 90° or 270°. The closest to native conformation generated using 30° (BCL) and 60° (BCL_60) binning differ by at least 0.4 Å.

Figure S2


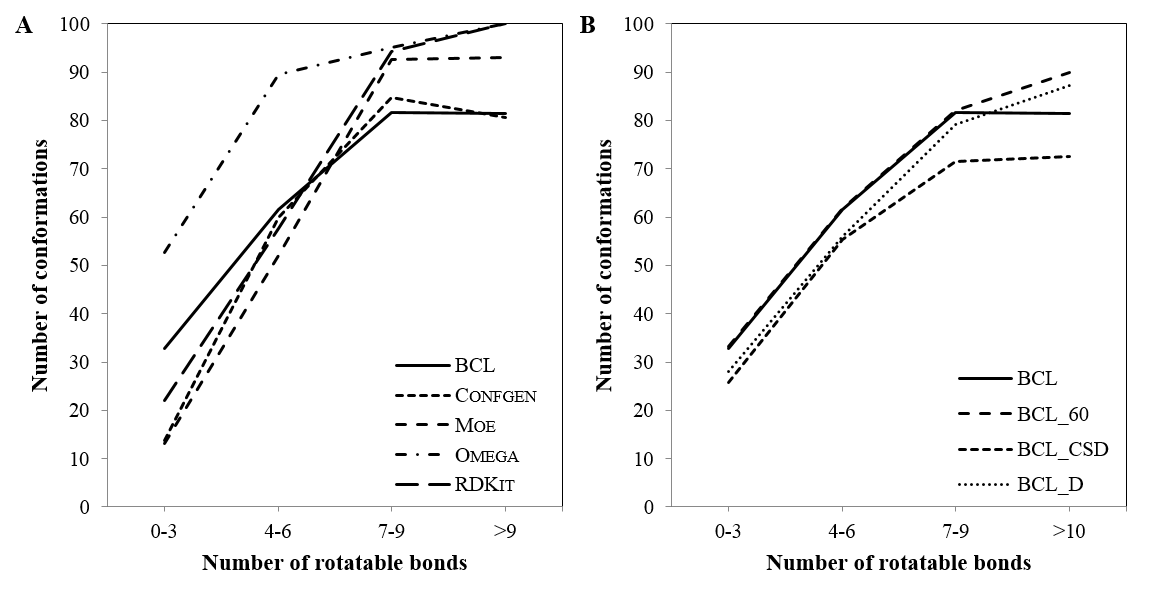


Figure S2. Average number of conformations generated by different methods as number of rotatable bonds increase. A) Comparison of commercially available methods to BCL. B) Comparison of different flavors of BCL.

Table S1 Optimization of BCL::Conf parameters using different number of iterations and temperature values. Optimization was done for better recovery of native conformations, fewer average number of conformations per molecule and computation time.

| **Itera**  **tions** | **T** | **Recovery%** | | | | | | | | | | **Average**  **number**  **of confo-**  **rmations** | **Time** |
| --- | --- | --- | --- | --- | --- | --- | --- | --- | --- | --- | --- | --- | --- |
|  |  | **0.25** | **0.5** | **0.75** | **1** | **1.25** | **1.5** | **1.75** | **2** | **2.25** | **2.5** |  |  |
| **200** | **1** | 11.46 | 36.36 | 62.45 | 76.68 | 86.17 | 91.30 | 96.44 | 99.21 | 100.00 | 100.00 | 52.40 | 1.6  s/mol |
|  | **2** | 11.46 | 36.76 | 59.68 | 78.26 | 87.35 | 92.49 | 98.02 | 99.60 | 99.60 | 100.00 | 57.28 |  |
|  | **3** | 10.67 | 37.15 | 61.66 | 79.45 | 88.93 | 93.28 | 97.63 | 99.60 | 99.60 | 100.00 | 60.27 |  |
|  | **4** | 11.07 | 37.94 | 61.66 | 77.08 | 86.17 | 93.28 | 96.84 | 100.00 | 100.00 | 100.00 | 61.75 |  |
| **250** | **1** | 9.88 | 37.55 | 63.24 | 76.68 | 86.17 | 92.49 | 95.26 | 98.02 | 98.81 | 99.21 | 58.00 | 1.9  s/mol |
|  | **2** | 10.28 | 35.57 | 61.66 | 76.68 | 85.38 | 91.70 | 96.05 | 98.81 | 98.81 | 99.21 | 64.81 |  |
|  | **3** | 11.86 | 36.36 | 62.45 | 79.05 | 88.54 | 91.70 | 96.05 | 98.42 | 98.81 | 99.21 | 66.83 |  |
|  | **4** | 11.86 | 39.92 | 61.66 | 78.26 | 88.14 | 92.49 | 96.44 | 98.81 | 99.21 | 99.21 | 66.43 |  |
| **300** | **1** | 10.67 | 37.15 | 63.64 | 79.05 | 88.93 | 94.07 | 97.23 | 99.21 | 99.60 | 99.60 | 63.23 | 2.2  s/mol |
|  | **2** | 11.46 | 37.55 | 64.82 | 79.05 | 87.35 | 91.70 | 96.84 | 98.42 | 98.81 | 99.21 | 68.36 |  |
|  | **3** | 11.07 | 37.94 | 67.59 | 79.05 | 88.54 | 91.70 | 97.23 | 98.42 | 98.42 | 98.81 | 70.75 |  |
|  | **4** | 12.65 | 39.92 | 65.22 | 80.24 | 87.75 | 92.89 | 96.84 | 100.00 | 100.00 | 100.00 | 71.75 |  |

**Protocol capture**

The protocol capture (Additional file 3) contains steps necessary to generate molecular conformations using BCL::Conf. The input parameter files and computational steps are necessary to make fragment library, rotamer library and using the rotamer library for conformational sampling. The final rotamer library and BCL::Conf executable can be downloaded at <http://www.meilerlab.org>. The commands required for generating rotamer library are provided in scripts which are included in the supplement.

| Step | Text | Commands | Comment |
| --- | --- | --- | --- |
| 1.Setup for running protocol capture | Download and unzip Additional file 3. The root directory is referred as **PATH** in the rest of the current table. | Download the BCL::Conf executable and bcl_license.txt (license file) at <http://www.meilerlab.org> and put it in the bin folder. |  |
| 1. Prepare the rotamer library from a given structure database. | If the structure database is large, jobs provided in the script will have to be split up. | Run the **PATH**/config/create_rotamer_library.sh script and provide the database as first parameter by using the following command –  /bin/bash **PATH/**config/ create_rotamer_library.sh [your database]  You can download the rotamer library obtained from CSD from <http://www.meilerlab.org> and keep it in **PATH**/bin to use it. | Input:  The structure database using which rotamer library will be created.  Output:  Rotamer library in the **PATH**/input directory is composed of three files and a directory :  rotlib.constitutions.txt.gz  rotlib.substructure.txt.gz  rotlib.configuration_mapping.txt.gz  directory - rotlib_conformations |
| 2. Generate conformation data for publication | Steps:  1. Generate conformations using methods of interest.  2. For each method, create a file containing rmsd of generated conformations to native conformation. Each line contains rmsd-to-native for conformations of a single molecule of the benchmark dataset.  3. Name the above file as vernalis_{method}_R.txt. An example file is vernalis_bcl_R.txt which contains rmsd-to-native values for the vernalis dataset. | BCL conformations were generated using –  **PATH**/bin/bcl-apps-static.exe molecule:ConformerGenerator -rotamer_library 'File(prefix=**PATH/**input/rotlib) –ensemble_filenames **INPUT** -top_models 100 -conformers_single_file **OUTPUT** –native_ensemble **NATIVE** –remove_h | Input:  **- INPUT** : **PATH**/input/{  zeroed_vernalis.sdf}  **- NATIVE** : **PATH**/input/{ native_vernalis.sdf}  Output:  **- OUTPUT** : **PATH**/input/{ vernalis_bcl_R.txt**}** |
| 3. Generate publication figures. | Steps:  1. Generate files containing rmsd-to-native data for each method and dataset as mentioned in step 2. | Execute script in **PATH/config** to generate plots :  **PATH/**config/generate_publication_figures.sh | Input:  **- PATH/**input/{all files listed below}  vernalis_bcl_R.txt,vernalis_confimport_R.txt,vernalis_confgen_R.txt,vernalis_dihedral_R.txt,vernalis_omega_R.tx,vernalis_rdkit_R.txt,  Output:  Image files in **PATH/**input  Comparison of closest to native conformer generated for each molecule in the dataset –  Files (example) :  vernalis_bcl_moe_comparison.txt (for all molecules),  vernalis_bcl_moe_comparison1.txt (molecules with rotatable bonds >0 and <4),  vernalis_bcl_moe_comparison1.txt (molecules with rotatable bonds >3 and <6), and so on |
| 3. Generate conformations by user defined parameters | An example command line to demonstrate user defined parameters that can be modified for conformational sampling | **PATH**/bin/bcl-apps-static.exe molecule:ConformerGenerator -rotamer_library 'File(prefix=**rotlib**)' –ensemble_filenames **INPUT** -temperature 3 -max_iterations 200 -conformation_comparer SymmetryRMSD 0.25 - top_models 100 -conformers_single_file **OUTPUT** |  |
