# Supplementary figures and images for: BCL::Conf: small molecule conformational sampling using a knowledge based rotamer library
Source: J Cheminform. 2015 Sep 30;7:47. doi: 10.1186/s13321-015-0095-1 (PMC4607025; doi:10.1186/s13321-015-0095-1)

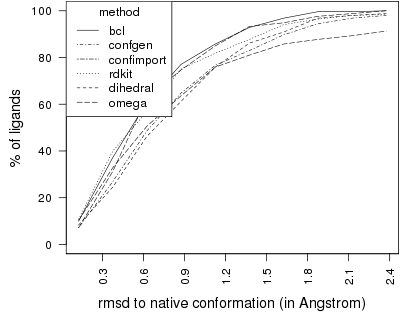

Supplement: Supplementary file 3 — Additional file 3. protocol capture files mentioned in Supplement.docx. [file 13321_2015_95_MOESM3_ESM.zip › protocol_capture/input/vernalis_recovery_programs.png]

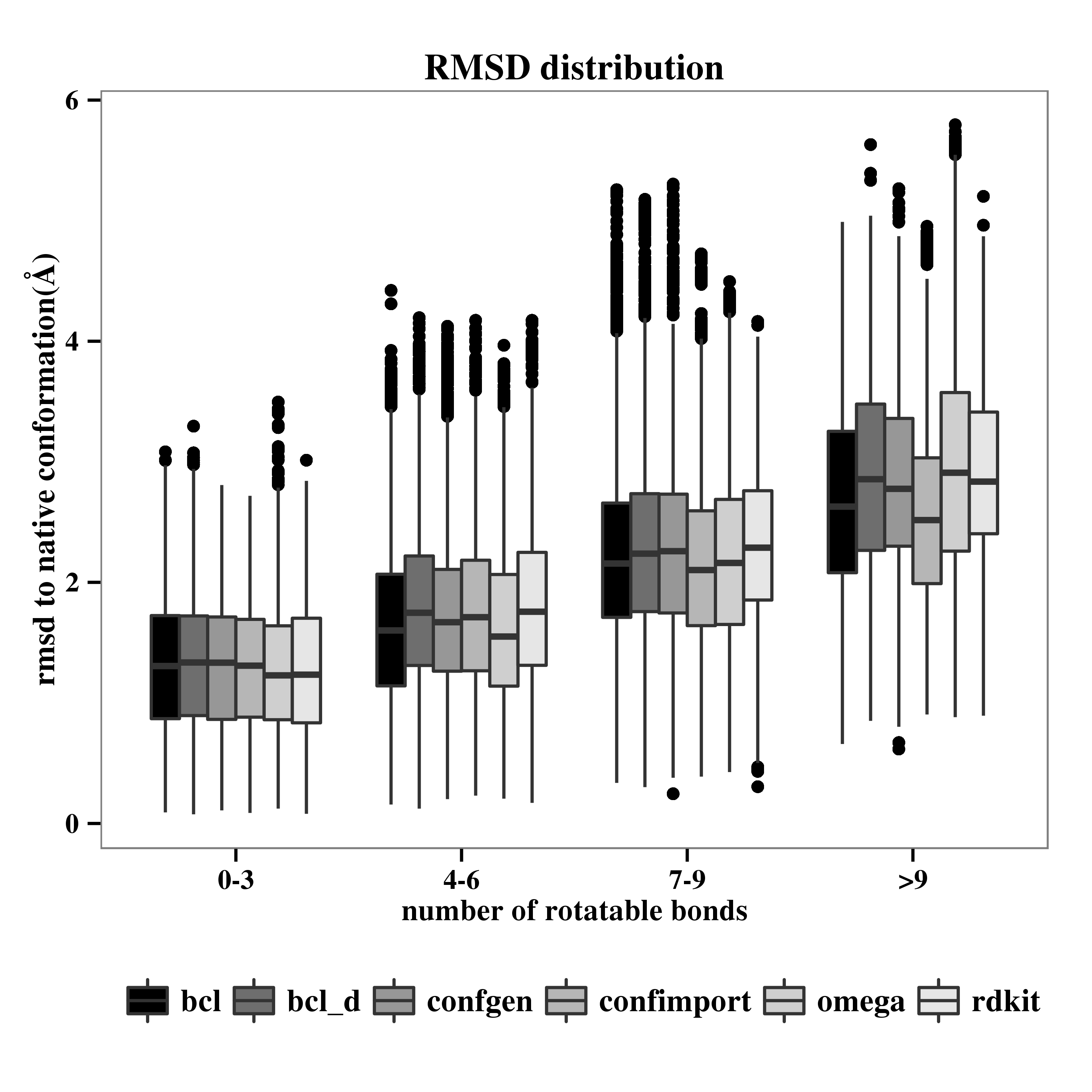

Supplement: Supplementary file 3 — Additional file 3. protocol capture files mentioned in Supplement.docx. [file 13321_2015_95_MOESM3_ESM.zip › protocol_capture/input/vernalis_confdistribution.tiff]

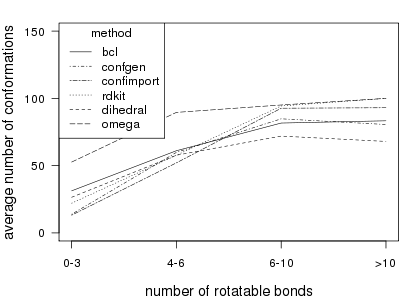

Supplement: Supplementary file 3 — Additional file 3. protocol capture files mentioned in Supplement.docx. [file 13321_2015_95_MOESM3_ESM.zip › protocol_capture/input/vernalis_average_confs.png]

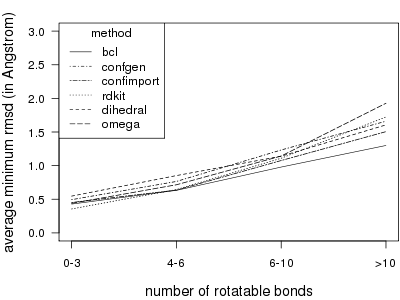

Supplement: Supplementary file 3 — Additional file 3. protocol capture files mentioned in Supplement.docx. [file 13321_2015_95_MOESM3_ESM.zip › protocol_capture/input/vernalis_average_min.png]
